# In Silico Prediction and Validation of CB2 Allosteric Binding Sites to Aid the Design of Allosteric Modulators

**DOI:** 10.3390/molecules27020453

**Published:** 2022-01-11

**Authors:** Jiayi Yuan, Chen Jiang, Junmei Wang, Chih-Jung Chen, Yixuan Hao, Guangyi Zhao, Zhiwei Feng, Xiang-Qun Xie

**Affiliations:** 1 Department of Pharmaceutical Sciences and Computational Chemical Genomics Screening Center, School of Pharmacy, University of Pittsburgh, Pittsburgh, PA 15261, USA; JIY106@pitt.edu (J.Y.); CHJ51@pitt.edu (C.J.); juw79@pitt.edu (J.W.); CHC327@pitt.edu (C.-J.C.); YIH83@pitt.edu (Y.H.); GUZ22@pitt.edu (G.Z.); 2 Department of Pharmaceutical Sciences and National Center of Excellence for Computational Drug Abuse Research, School of Pharmacy, University of Pittsburgh, Pittsburgh, PA 15261, USA

**Keywords:** cannabinoid receptor 2, allosteric binding site, positive allosteric modulators, negative allosteric modulators

## Abstract

Although the 3D structures of active and inactive cannabinoid receptors type 2 (CB2) are available, neither the X-ray crystal nor the cryo-EM structure of CB2-orthosteric ligand-modulator has been resolved, prohibiting the drug discovery and development of CB2 allosteric modulators (AMs). In the present work, we mainly focused on investigating the potential allosteric binding site(s) of CB2. We applied different algorithms or tools to predict the potential allosteric binding sites of CB2 with the existing agonists. Seven potential allosteric sites can be observed for either CB2-CP55940 or CB2-WIN 55,212-2 complex, among which sites B, C, G and K are supported by the reported 3D structures of Class A GPCRs coupled with AMs. Applying our novel algorithm toolset-MCCS, we docked three known AMs of CB2 including Ec2la (C-2), trans-β-caryophyllene (TBC) and cannabidiol (CBD) to each site for further comparisons and quantified the potential binding residues in each allosteric binding site. Sequentially, we selected the most promising binding pose of C-2 in five allosteric sites to conduct the molecular dynamics (MD) simulations. Based on the results of docking studies and MD simulations, we suggest that site H is the most promising allosteric binding site. We plan to conduct bio-assay validations in the future.

## 1. Introduction

Cannabinoid receptor type 1 (CB1) and type 2 (CB2) belong to G protein coupled receptors (GPCRs), which are the largest and the most diverse membrane protein family in the human genome [1]. Like other GPCRs, CB1 and CB2 are the potential therapeutic targets of many diseases, such as pain, obesity, neuroinflammation, immune suppression, cancer and osteoporosis [2,3,4]. CB1 receptors are found throughout the body and widely distributed in the central nervous system (CNS), while CB2 receptors are expressed mostly in the peripheral immune system. Although CB2 has recently been identified to express in the brain, the expression level is much lower than CB1 [5]. CB1 is mainly responsible for psychiatric effects such as antiemetic and analgesic actions of tetrahydrocannabinol (THC) and several metabolic processes. CB2 [1,2,3,4] is mainly associated with immune suppression, apoptosis and cell migration and thus gradually becoming a therapeutic target for immunomodulation, inflammatory and neuropathic pain, neurodegenerative disorders, neuroinflammation, fibrotic condition and cancer. Since CB1 is majorly expressed in the CNS, activation of CB1 may precipitate psychosis and panic, while inhibition may cause depression as well as anxiety [6]. Thus, compounds or modulators that can selectively bind to the CB2 receptor may have potential to produce therapeutic effects and avoid CNS side effects.

Compounds targeting at CB2 can be divided into orthosteric ligands and allosteric modulators (AMs) based on their mechanism of action. Orthosteric ligands bind to the binding site of endogenous molecule(s) and directly cause physiological responses. AMs bind to the additional binding site(s) of the receptor and affect the function of dependent orthosteric ligand(s). According to the pharmacological effects, AMs are divided into positive allosteric modulators (PAMs), negative allosteric modulators (NAMs) and silent allosteric modulators (SAMs). PAMs can increase the functional response of the dependent orthosteric ligand, while NAMs can inhibit the related functional response. SAMs do not affect the orthosteric ligand [7] but can prevent the binding of PAMs and NAMs [1,3]. Compared to the orthosteric ligands, compounds targeting on allosteric binding sites show higher selectivity because the allosteric binding sites are less conserved than orthosteric binding sites in a protein family [8]. Besides, AMs play a role in cooperating with orthosteric ligand to stabilize different conformational states of GPCRs. AMs can reach “effect ceiling” which improves the safety of target in overdose situations. Therefore, more and more AMs of GPCRs have been discovered as the potential drugs [5]. For examples, Ticagrelor is an anti-thrombosis drug approved by FDA, which acts as an allosteric antagonist and targets on P2Y receptor [9]. Maraviroc is a NAM of CC chemokine receptor type 5 (CCR5), which is approved for the treatment of HIV infection [10]. Another example is Plerixafor, a NAM of CXC chemokine receptor type 4 (CXCR4), approved by FDA for the treatment of bone marrow transplantation [11,12].

We recently summarized 11 PDB files [13] containing the crystal structures of reported Class A GPCRs (including Alpha, Delta and Gamma sub-branches). We also discussed the reported allosteric binding sites in this study and found that four allosteric binding sites are more typical than others. Meanwhile, due to the limited number of reported crystal structures of receptor–AM, the conclusion for each allosteric binding site may only indicate the possible binding features of the receptor–AM complex.

As we know, the structure of CB1 is the most similar to that of CB2 among GPCRs. There are seven X-ray crystal or cryo-EM structures of CB1 in the Protein Data Bank (https://www.rcsb.org accessed on 1 January 2022). Among them, one complex of CB1-agonist-NAM has been reported. In the crystal structure of CB1-CP55940-ORG27569 [14], ORG27569 is a NAM of CB1 and CP55940 is an orthosteric agonist of CBs (refers to CB1 and CB2) [14]. Meanwhile, only four complexes of CB2 are available, including two complexes of CB2-agonist [15], a 3D cryo-EM structure of CB2-WIN 55,212-2-Gi signaling complex [2] and a structure of CB2 coupled with antagonist [16]. In addition, several AMs have been discovered for CB2, including pepcan-12 [17], compound Ec2la (C-2) [18], trans-β-caryophyllene (TBC) [19] and cannabidiol (CBD) [19]. However, we still do not know how these AMs bind to CB2, and there is no X-ray crystal or cryo-EM structure of CB2-orthosteric ligand(s) coupled with the modulator. All the missing information seriously hinders the drug discovery and development process of CB2 allosteric modulators.

In the present work, we mainly focused on investigating the potential allosteric binding site(s) of CB2. We first predicted the allosteric cavities of CB2 in the presence of CP55940 or WIN 55,212-2 using three different algorithms. We then docked three reported AMs into each predicted site of CB2 and analyzed the detailed interactions and energy distribution between AMs and involved residues. By selecting the binding pose of C-2 with the lowest total energy contribution in each allosteric site, we sequentially carried out the MD simulations for the complexes of CB2-CP55940-C-2 coupled with Gi protein. Based on our findings, we suggest that site H is the most promising allosteric binding site of C-2.

## 2. Results

### 2.1. Overview of Seven Predicted Allosteric Binding Sites in CB2-WIN 55,212-2 or CB2-CP55940 Complex

Three different algorithms including CavityPlus, Protein Allosteric and Regulatory Sites (PARS) and Sybyl-X were used to predict the potential allosteric binding sites of CB2 using both the cryo-EM structure of CB2-WIN 55,212-2 [2] and the modeled complex of CB2-CP55940 [14].

**Figure 1:**
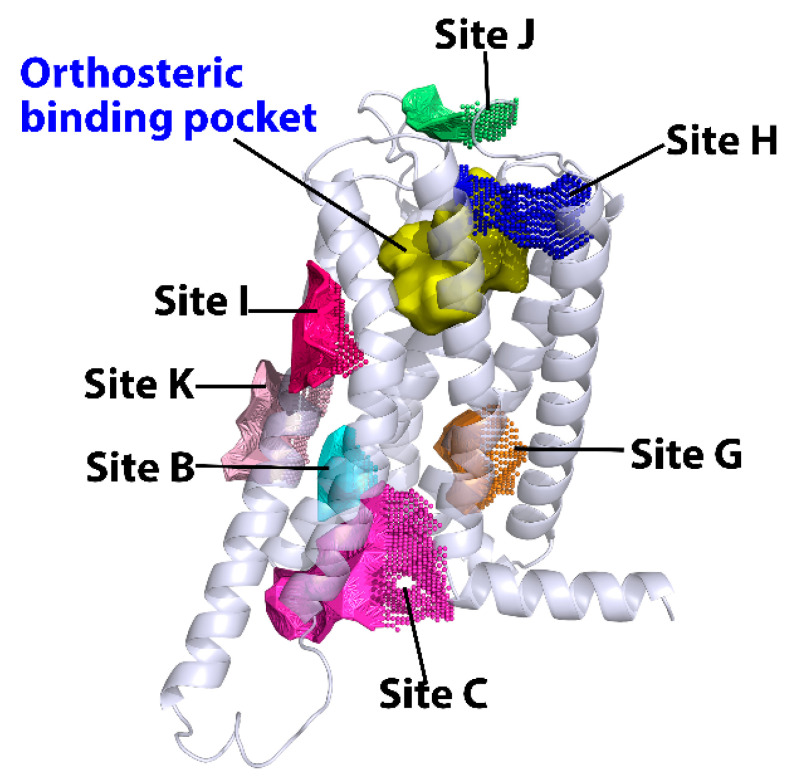
Predicted allosteric binding sites of CB2 by CavityPlus, Protein Allosteric and Regulatory Sites (PARS) and Sybyl-X. The orthosteric binding site was highlighted in yellow surface, while predicted allosteric sites are highlighted in dots.

As shown in Figure 1, there were a total of seven promising allosteric binding sites predicted by the algorithms. Among them, four allosteric sites were supported by the reported allosteric binding pockets in Class A GPCRs, including sites B, C, G and K.

Site B is located between the TM3 and TM4 and is close to the intracellular loop 2 (ICL2), which is supported by two 3D structures, including the complex of beta-2 adrenergic receptor (ADRB2)-orthosteric agonist BI167107-PAM Compound-6FA (Cmpd-6FA) reported by Liu and co-workers [20] (PDB:6N48), and the complex of free fatty acid human receptor 1 GPR40 (FFAR1)-orthosteric partial agonist MK-8666-PAM compound-AP8 (Cmpd-AP8, PDB:5TZY). After aligning those two crystal structures with CB2 receptor, we found that Cmpd-6FA locates exactly at the same position as site B. The carboxylate group of Cmpd-AP8 inserts in a polar cavity of FFAR1 formed by TM3, TM4 and ICL2, which is the same position as site B in Figure 1 [21]. Recently, the cryo-EM structures of dopamine D1 receptor (DRD1)-orthosteric agonist dopamine-PAM LY3154207 (PDB: 7CKZ) has been reported by Xiao and co-workers. The binding site of LY3154207 is created by ICL2, TM3 as well as TM4, which is also the same position as site B [22].

Site C is located within the receptor’s helical bundle, TMs 1–3, TMs 6–7 and helix 8, on the intracellular side of CB2 receptor. It is accessible from cytoplasm, and it overlaps with the Gα binding site. This site is supported by the crystal structure (PDB:5T1A) of CC chemokine receptor type 2 (CCR2)-orthosteric antagonist BMS-681-NAM CCR2-RA-[R] published by Zheng and colleagues [23]. The position of site C has also demonstrated by other crystal structure of CC chemokine receptor type 9 (CCR9)-NAM vereirnon (PDB:5LWE) [24] as well as the complex of ADRB2-orthosteric antagonist Carazolol-NAM Compound-15PA (Cmpd-15PA) (PDB:5X7D) [25]. All these NAMs mentioned above locate at the intracellular site of their receptors and appeared to sterically interfere with the G-protein binding to their receptors [23,24,25].

Moreover, site G is at the extrahelical site in the inner part of the membrane, which is located at the bottom of TMs 1, 2, 3 and 4. Site G is supported by the crystal structure of CB1-orthosteric antagonist CP55940-NAM ORG27569 (PDB:6KQI) [14].

The location of site K between TM3 and TM5 is supported by the 3D structure of ADRB2-orthosteric antagonist alprenolol-NAM AS408 (PDB:6OBA). They found that the well-defined Fo–Fc electron densities for AS408 is on the membrane-facing surfaces of TM3 and TM5, but not in the extracellular vestibule [26]. Another supporting 3D structure of site K is C5a anaphylatoxin chemotactic receptor 1 (C5aR1)-NAM NDT9513727 (PDB:5O9H) [27]. By aligning the crystal structure of C5aR1 human receptor with ADRB2 structure (PDB:6OBA), we observed that NDT9513727 binds to the same region as the location of NAM-AS408 in ADRB2 human receptor, which is consistent with the site K in Figure 1.

For other three novel allosteric binding sites, site J is located at the top of TMs 3–7, site I is near TMs 5 and 6, while site H is close to the orthosteric binding site that formed by the residues in TMs 1, 2 and 7. The promising binding residues involved in these potential allosteric binding sites will be discussed in the following sections.

### 2.2. Detailed Binding Poses and Interactions of C-2, TBC and CBD in Sites B, K and C of CB2

Up to date, C-2 (PAM) [18], TBC (NAM) [28] and CBD (NAM) [19] are the reported CB2 small-molecule AMs. As shown in Table 1, we first docked these three AMs into seven predicted allosteric sites of the CB2 in the presence of agonist-CP55940. The binding pose of CP55940 in CB2 was identical to that in CB1. We also scored the crystal structures of other GPCRs that supported our predicted binding site and made comparisons with the detailed residue energy contribution of CB2.

**Figure 2:**
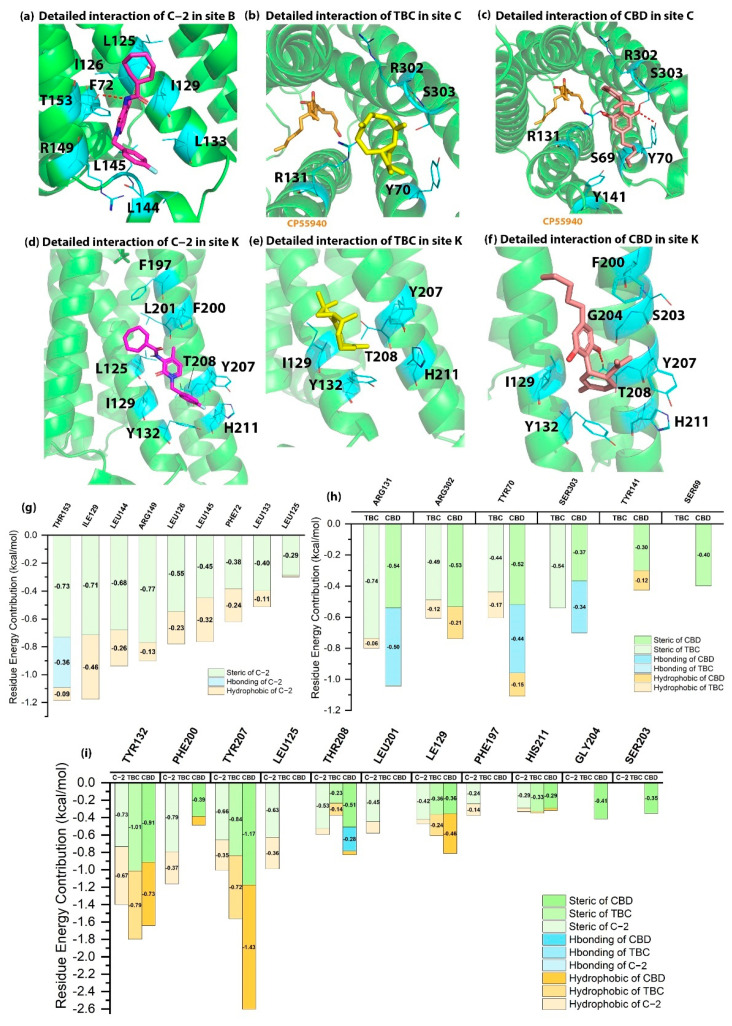
The detailed interactions and residue energy contribution of C-2, TBC and CBD in sites B, C and K. (**a**) The detailed binding pose of C-2 in site B. (**b**) The detailed binding pose of TBC in site C. (**c**) The detailed binding pose of CBD in site C. (**d**) The detailed binding pose of C-2 in site K. (**e**) The detailed binding pose of TBC in site K. (**f**) The detailed binding pose of CBD in site K. (**g**) The residue energy contribution of key residues for C-2 in site B. (**h**) The comparisons of residue energy contribution of key residues for TBC and CBD in site C. (**i**) The comparisons of residue energy contribution of key residues for C-2, TBC and CBD in site K.

As shown in Figure 2, we docked C-2 into site B, TBC/CBD into site C, and all three AMs into site K. Specially, C-2 formed a hydrogen bond with T153^4.45^ and hydrophobic interactions with I129^3.48^, R149^4.41^ and L145^ICL2^ in site B. These important residues involved in the binding of C-2 were supported by the crystal structures of human FFAR1 receptor and human ADRB2 receptor [20,21]. In our scoring results, Cmpd-6FA, a PAM locates in site B of ADRB2, interacts with F133^3.52^ and L144^ICL2^ via hydrophobic and steric interactions and forms a strong hydrogen bond with K149^4.41^, which are consistent with the literature [20]. To study the detailed interaction of AMs in site B, we also docked TBC/CBD into site B. We found that I129^3.48^, L145^ICL2^, R149^4.41^ and T153^4.45^ were the residues with high energy contribution for TBC and CBD. Both arginine and lysine were basic residues with positively charged side chains, so the interactions between K149^4.41^ (ADRB2) and AMs were similar to that of R149^4.41^ (CB2). We predicted that T153^4.45^, I129^3.48^, R149^4.41^ and L145^ICL2^ are potential key residues in site B.

In site C, TBC formed the steric interactions with R131^3.50^, R302^7.56^, Y70^2.40^ and S303^8.47^, while CBD formed hydrogen bonds with Y70^2.40^, R131^3.50^ and S303^8.47^. The key residues for the binding of TBC and CBD are supported by the crystal structures of CCR2 human receptor, CCR9 human receptor and ADRB2 human receptor [23,24,25]. For example, Cmpd-15PA, a NAM of ADRB2, forms hydrogen bonds with R63^ICL1^, D331^8.49^, S329^8.47^ and T274^6.36^ and hydrophobic interactions with Y326^7.53^, F332^8.50^ and L64^ICL1^. We found that at position 8.47, serine was the only overlapped residue that formed the same interaction between CB2 and ADRB2. However, the binding poses of NAMs in CCR9, CCR2 and ADRB2 were much closer to TMs 1, 7 and helix 8 compared to the binding poses of TBC/CBD in CB2 due to the spatial block by Y70^2.40^. Taking all the supporting literature and the binding site of Gi protein into consideration, we inferred that C-2 (PAM) was less possible to bind at site C.

Site K is adjacent to site B. The distance between the centers of two pockets is approximately 12 angstroms, of which the boundaries between them is about 3.6 angstroms. Our predictions are supported by 3D structures of other GPCRs as mentioned previously. We docked C-2, TBC and CBD into site K of CB2 to study their detailed interactions. As shown in Figure 2d–f, Y207^5.56^ and Y132^3.51^ contributed hydrophobic and steric interactions to the binding of C-2, TBC and CBD. In addition, F200^5.49^, L125^3.44^ and I129^3.48^ contributed to the steric and hydrophobic interactions among three AMs, which are supported by crystal structures of ADRB2 and C5aR1 [26,27]. In ADRB2, C125^3.44^, V129^3.48^ and V210^5.49^ create the binding site which is consistent with site K. Especially, at the position of 5.49, V210 has high energy contribution to the binding of AS408. Meanwhile, W213^5.49^ in C5aR1 also significantly contributes to the binding of NDT9513727 via its indole ring. Based on our docking results, F200^5.49^ in CB2 possesses a benzene ring, suggesting that at position 5.49, F200 may be a critical residue in site K of CB2.

### 2.3. Detailed Binding Poses and Interactions of C-2, TBC and CBD in Site G of CB2

**Figure 3:**
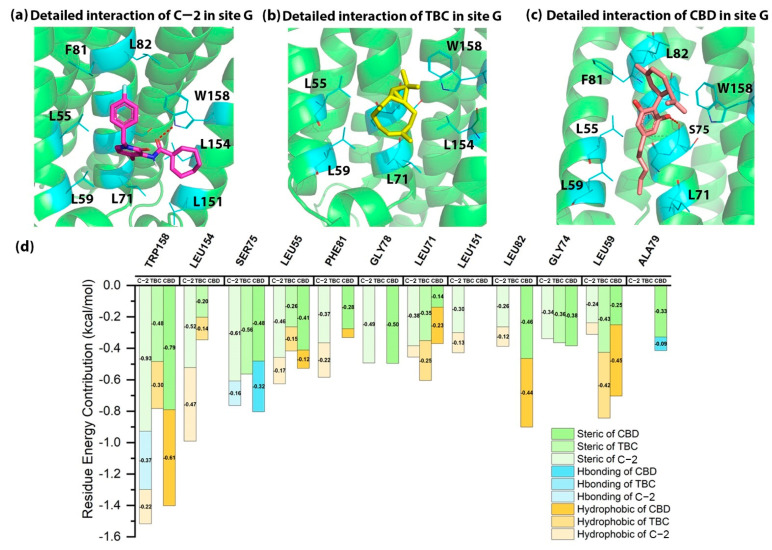
The detailed interactions and residue energy contribution of C-2, TBC and CBD in site G. (**a**) The detailed binding pose of C-2 in site G. (**b**) The detailed binding pose of TBC in site G. (**c**) The detailed binding pose of CBD in site G. (**d**) The comparisons of residue energy contribution of the key residues for these AMs in site G.

As shown in Table 1, the docking energies of C-2, TBC and CBD in site G were −6.68, −5.42 and −5.89 kcal/mol. As shown in Figure 3d, W158^4.50^ was the critical residue that contributed greatly to the binding of AMs in site G. W158^4.50^ was predicted to form a hydrogen bond with C-2, hydrophobic interactions with TBC and CBD, and the steric interactions with all three AMs. Moreover, our results also showed that S75^2.45^ formed a hydrogen bond with CBD and steric interaction with all AMs. In addition, L55^1.54^ formed steric interaction with C-2 as well as CBD. L154^4.46^ contributed to the hydrophobic and steric interactions with C-2 in site G.

The key residues involved in the binding of C-2, TBC and CBD were supported by the crystal structure of CB1-orthosteric CP55940-NAM ORG27569 as mentioned previously. For example, I245^4.54^, T242^4.51^, C238^4.47^, V161^2.48^, F237^4.46^ and W241^4.50^ of CB1 contributed to binding of ORG27569. Among those residues, W241^4.50^(CB1) and W158^4.50^(CB2) are highly conserved in class A GPCRs [14], and both are predicted to have high energy contribution to the binding of AMs in CBs. Thus, we suggested that W158^4.50^ and L154^4.46^ may be the key binding residues in site G.

### 2.4. Detailed Binding Poses and Interactions of C-2, TBC and CBD in Site H of CB2

**Figure 4:**
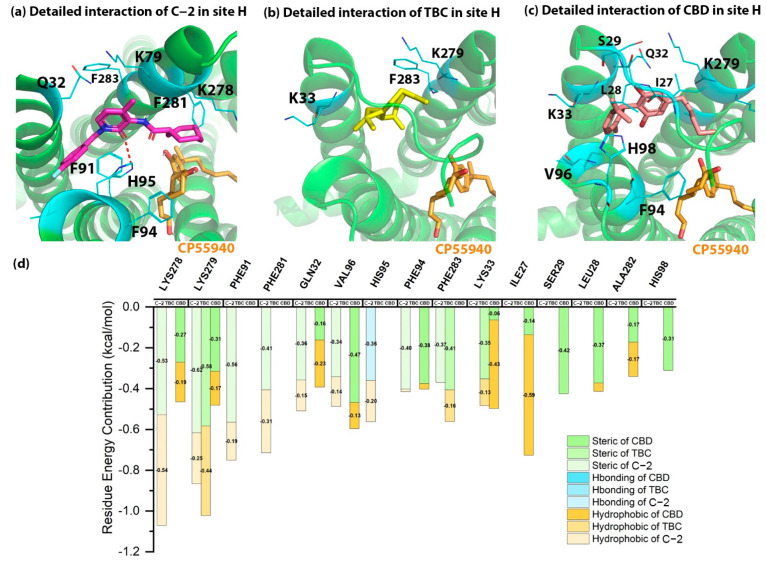
The detailed interactions and residue energy contribution of C-2, TBC and CBD in site H. (**a**) The detailed binding pose of C-2 in site H. (**b**) The detailed binding pose of TBC in site H. (**c**) The detailed binding pose of CBD in site H. (**d**) The comparisons of residue energy contribution of these AMs in site H.

As shown in Table 1, the docking energies of C-2, TBC and CBD in site H were −7.20, −2.79 and −4.21 kcal/mol respectively. The lower docking score for TBC was due to its smaller structure. When looking into the detailed interactions of C-2, TBC and CBD in site H, there were steric and hydrophobic interactions along with only one hydrogen bonding. As shown in Figure 4, H95^2.65^ formed a hydrogen bond with C-2. K278^7.31^ and K279^7.32^ contributed the highest binding energy to the binding of AMs in CB2. For example, K278^7.31^ formed steric and hydrophobic interactions with C-2, while K279^7.32^ contributed to the steric interaction for the binding of all AMs and a hydrophobic interaction to that of TBC. F91^2.61^ and F281^7.34^ made contributions to the binding of C-2 via a steric interaction in site H.

Up to date, there is no available crystal or cryo-EM structure of receptor–AM to support this binding site. However, this allosteric site is supported by computational and biological validation, which is about the potential docking pose of D3R-selective antagonist R-22 in the crystal structure of human dopamine D3 receptor (D3R) [29]. For example, the authors suggested that the R-22 might extend to the binding site formed by TMs 1-2-7 region of D3R with eticlopride and have interactions with some key residues, such as E90^2.65^. Based on the structure of eticlopride and R-22 in D3R, Lane and co-workers [30] predicted another potential allosteric binding site that extends toward ECL2, formed by TM1, TM2 and TM7, which is at the same location of site H in Figure 1. The authors then conducted the virtual screening and identified some hit compounds as the AMs in their work. In addition, our previous work [31] also supported the binding site of site H, in which we docked the bitopic ligand-SB269652 into dopamine 2 receptor (D2R). Our docking results indicated that the allosteric fragment of SB269652 bound to the allosteric binding site formed by TMs 1-2-7.

Although CB1 has the most similar sequence to CB2, it has not been demonstrated by X-ray crystal or cryo-EM structures that CB1 has an allosteric binding site in the same position as site H of CB2. Based on the structural alignment and our docking results, K278^7.31^ and K279^7.32^ of site H in CB2 refer to K376^7.31^ and T377^7.32^ in CB1, respectively. One of the key residue is the histidine at the position of 2.65 at CB2 (H95) and CB1 (H178), which is able to form hydrogen bonds with ligands.

### 2.5. Detailed Binding Poses and Interactions of C-2, TBC and CBD in Sites I and J of CB2

**Figure 5:**
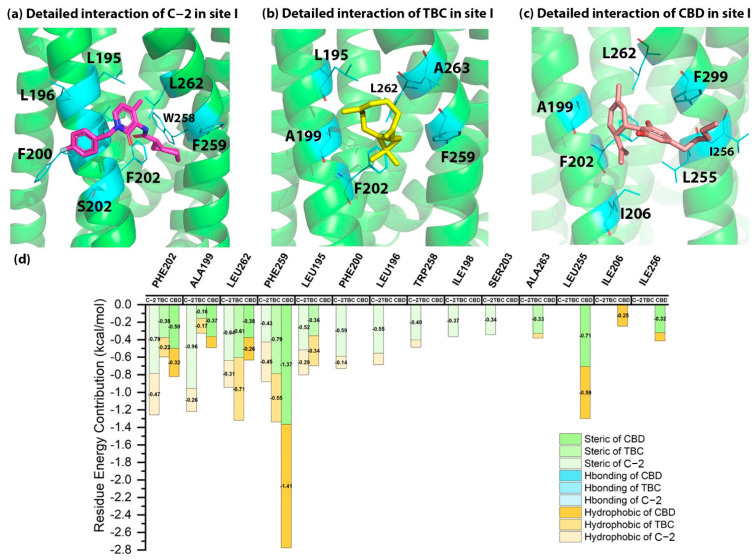
The detailed interactions and residue energy contribution of C-2, TBC and CBD in site I. (**a**) The detailed binding pose of C-2 in site I. (**b**) The detailed binding pose of TBC in site I. (**c**) The detailed binding pose of CBD in site I. (**d**) The comparisons of residue energy contribution of binding residues for these AMs in site I.

The docking energies of C-2, TBC and CBD in site I were −7.31, −6.39 and −5.92 kcal/mol, indicating the potential of site I in CB2. Comparing the detailed interactions of C-2 in site I, several strong steric and hydrophobic interactions were observed in the binding site. As illustrated in Figure 5, C-2 had a hydrophobic interaction with F202^5.51^ as well as the strong steric interaction with F202^5.51^ and A199^5.48^. For TBC, it formed a steric interaction with F259^6.49^ and a hydrophobic interaction with L262^6.52^. In addition, CBD interacted with L255^6.45^ via strong steric interaction and contacted F259^6.49^ via a strong hydrophobic interaction (−1.4 kcal/mol). Since the residue energy contributions in site I were different among C-2, TBC and CBD, it was hard to say which residues were more important for the binding of AMs in CB2. But as for C-2, F202^5.51^, A199^5.48^ and L262^6.52^ were the potential key residues that contributed mostly to the binding with CB2 in site I.

**Figure 6:**
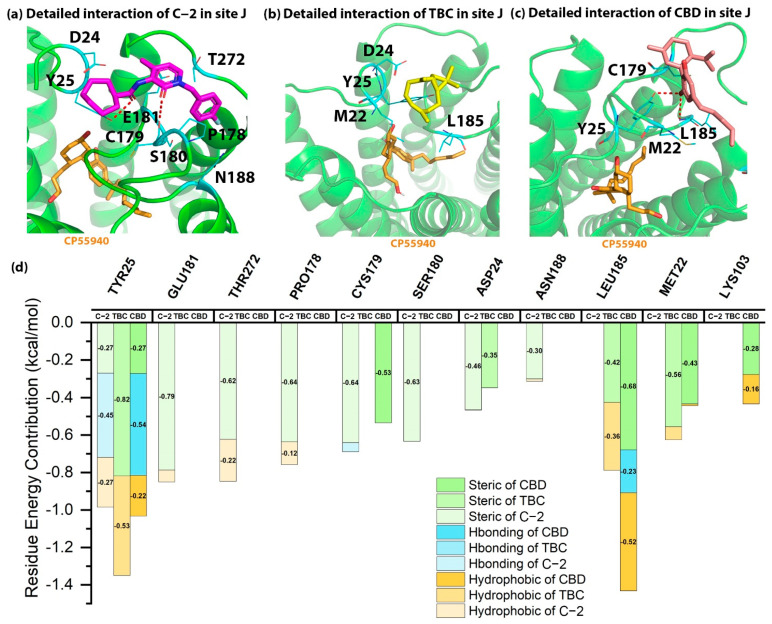
The detailed interactions and residue energy contribution of C-2, TBC and CBD in site J. (**a**) The detailed binding pose of C-2 in site J. (**b**) The detailed binding pose of TBC in site J. (**c**) The detailed binding pose of CBD in site J. (**d**) The comparisons of residue energy contribution of the binding residues for these AMs in site J.

As illustrated in Table 1, the docking scores of three AMs in site J were not as good as those in sites B, G and I. As shown in Figure 6, T25 contributed greatly to the binding of C-2, TBC and CBD, including the hydrogen bonds with C-2 as well as CBD and the hydrophobic/steric interactions with TBC. In addition, E181 in site J also interacted with C-2 via a strong steric interaction, while L185 contacted CBD via steric and hydrophobic interactions. For site J, our docking results showed that T25 might be an important residue in this binding site.

### 2.6. MD Simulations for C-2 in Five Predicted Allosteric Binding Sites

**Figure 7:**
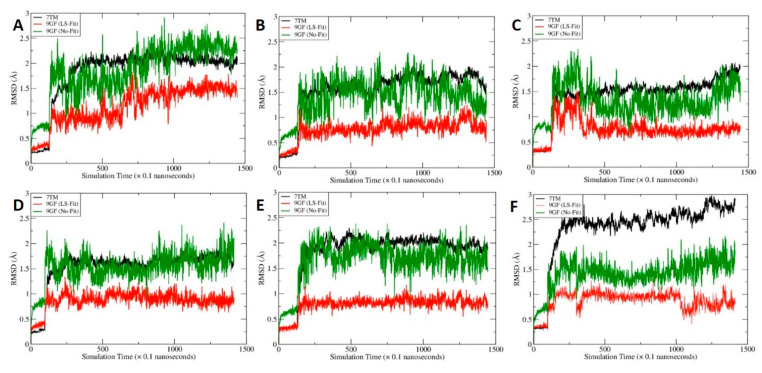
The time course of root–mean–square deviations (RMSD) of mainchain atoms of the transmembrane domains (7TM, black), heavy atoms of CP55940 after least-square (LS) fitting (9GF, red) and heavy atoms of CP55940 without fitting (9GF, green). To calculate the No-Fit RMSDs for CP55940, the mainchain atoms of 7TM were first aligned and the resulting translation–rotation matrix were then applied to the ligand. After the coordinate transformation, the RMSDs were calculated directly. (**A**) CP55940/CB2 without C-2 binding, (**B**) site B, (**C**) site G, (**D**) site H, (**E**) site I and (**F**) site J.

**Figure 8:**
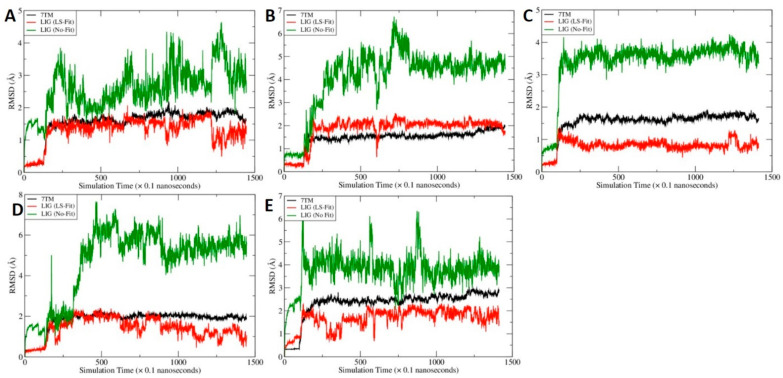
The time course of root–mean–square deviations of mainchain atoms of the transmembrane domains (7TM, black), heavy atoms of C-2 after least-square (LS) fitting (LIG, red) and heavy atoms of C-2 without fitting (LIG, green). To calculate the No-Fit RMSDs for C-2, the mainchain atoms of 7TM were first aligned and the resulting translation–rotation matrix were then applied to the ligand. After the coordinate transformation, the RMSDs were calculated directly. (**A**) site B, (**B**) site G, (**C**) site H, (**D**) site I and (**E**) site J.

Molecular dynamics (MD) simulations were performed for the five promising binding sites, namely, site B, site G, site H, site I and site J. For each system, 250 snapshots were evenly collected from the 130-ns MD trajectory after the system reached equilibrium for post-MD analysis. As shown in Figure 7 and Figure 8, all the MD systems reached equilibrium after 20 ns.

The calculated molecular mechanics/Poisson-Boltzmann surface area-WSAS (MM-PBSA-WSAS) free energies for the CB2/CP55940/C-2 complex were summarized in Table 2. Site H has the best free energy of −7321.31 kcal/mol, which is significantly lower than other binding sites, suggesting site H is the most possible binding site for the allosteric binding. We further calculated the binding free energies between CB2 and C-2 (Table 3) as well as CB2 and CP55940 (Table 4). As shown in Table 3, the binding between CB2 and C-2 are weak for all the sites except for site H, for which the calculated MM-PBSA-WSAS binding free energy is −34.12 kcal/mol. Interestingly, the binding between CP55940 and CB2 is enhanced for most binding sites as shown in Table 4.The MM-PBSA-WSAS binding free energy, −27.39 kcal/mol when C-2 is absent from the complex, can be considered as the reference to predict if C-2 is a PAM or NAM. Considering it is the free energy of the complex that really matters, we mainly focused on site H. With C-2 residing at site H, the MM-PBSA-WSAS of CP55940 binding, −26.09 kcal/mol, is slightly worser than the control suggesting C-2 is a NAM. However, site H is adjacent to the orthosteric site and C-2 and CP55940 has a favorable interaction between them. Thus, C-2 should be considered as a part of receptor when calculating the binding free energy of CP55940. With C-2 being considered as a part of receptor (labeled as H* in Table 4), the calculated MM-PBSA-WSAS binding free energy is −32.04 kcal/mol, about 4.6 kcal/mol more potent than the control. Thus, C-2 is a PAM under this circumstance.

**Figure 9:**
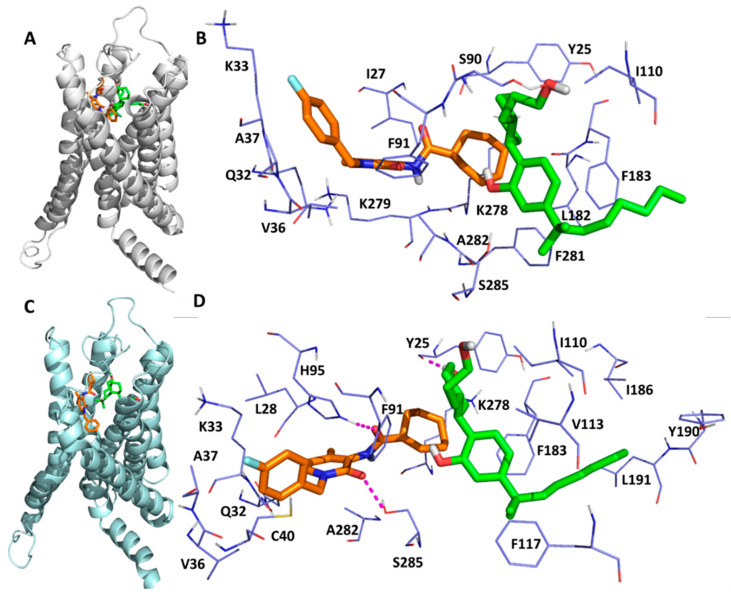
The detailed interactions between C-2, CP55940 and CB2 receptor where C-2 resides in site H. (**A**) The crystal structure of CP55940/CB2 with C-2, the PAM binding at site H. (**B**) The CB2 residues surrounding CP55940 and C-2. (**C**) The representative MD structure (the one that is structurally most similar to the average structure). (**D**) The residues surrounding CP55940 and C-2 in the representative MD structure. Two hydrogen bonds are formed between C-2 and CB2 residues (H95^2.65^ and S285^7.38^), and one hydrogen bond is formed between CP55940 and Y25. The hydrogen bonds are shown as magenta dashed lines.

The C-2 binding mode before and after MD simulations is illustrated by Figure 9A,C. Note that the crystal structure and the representative MD conformation were aligned so that one can compare them directly. We found that CP55940 has insignificant changes before and after MD simulations (Figure 7D and Figure 9A,C), while C-2 undergoes significant changes (Figure 8C and Figure 9A,C). The surrounding residues shown in Figure 9B are for the crystal structure and docking conformation, whereas those in Figure 9D are for the representative MD conformation. Note that CP55940 forms a hydrogen bond with T25, and C-2 forms two hydrogen bonds with H95^2.65^ and S285^7.38^. Those residues could be candidates for the mutagenesis experiment to confirm the proposed binding mechanism, i.e., C-2 binds at site H and it can boost the activity of CP55940 binding to CB2.

## 3. Materials and Methods

### 3.1. 3D Structures of CB2

The cryo-EM structure of CB2 coupled with agonist-WIN 55,212-2 [2] was collected from the Protein Data Bank (https://www.rcsb.org accessed on 1 January 2022) [32,33]. Since there is no available crystal or cryo-EM structure of CB2-CP55940, we built this complex using the molecular docking approach. CP55940 is a non-selective agonist of CB1 and CB2 [34]. The crystal 3D structure of CB1-CP55940 [14] was used to select the most appropriate binding pose of CP55940 in CB2.

### 3.2. Predictions of Allosteric Binding Sites of CB2

Three algorithms or software were used to predict the potential allosteric binding sites of CB2, including the CavityPlus (http://www.pkumdl.cn:8000/cavityplus/index.php accessed on 1 January 2022) [35], Protein Allosteric and Regulatory Sites (PARS) (http://bioinf.uab.cat/cgi-bin/pars-cgi/pars.pl accessed on 1 January 2022) [36], and Sybyl-X 1.3 (SYBYL-X Software|Certara).

In CavityPlus, we adopted Cavity module and CorrSite 2.0 submodule. First, we upload CB2 structure of PDB:6PT0 by removing WIN55,212-2. Based on the structural geometry-based method, the Cavity module can detect the potential binding sites on a given protein structure, which were then ranked by their ligandability and druggability quantitatively assessed by CavityScore and CavityDrugScore respectively. Next, CorrSite 2.0 is adopted to further identify the allosteric sites with higher potentiality among all the predicted cavities. We defined the orthosteric sites by uploading a custom pocket generated by PDB:6PT0. Based on the hypothesis that there was a high correlation between the motions of orthosteric and allosteric sites, CorrSite 2.0 calculates the motion correlation between the allosteric binding sites and the known orthosteric binding site. We selected the sites with z-score higher than 0.5.

In PARS, we uploaded the complex of CB2-CP55940/CB2-WIN55,212-2 and the potential binding sites were generated with overall flexibility value and structural conservation. In Sybyl-X, we adopted the Surflex-Dock module, uploaded the complex of CB2-CP55940/CB2-WIN55,212-2, and defined the binding sites with multi-channel surface mode. We set “Threshold” as 0.50 and “Bloat” as 0. Then, we aligned the pockets generated by three algorithms and tools, selected the binding sites that detected in all two complexes by at least two methods, and compared them to the X-ray crystal and cryo-EM structures of GPCRs.

### 3.3. Molecular Complex Characterizing System (MCCS)

Here we applied MCCS [37] to prepare the complexes and calculate the residue energy contribution. Firstly, each PDB was divided into two parts: an orthosteric ligand PDB and a protein PDB. A protein PDB may contain both a protein and an allosteric modulator, or just the protein structure. Chimera (version 1.15) [38] was used to fix residues with an incomplete side chain in protein PDBs. Specifically, Chimera scanned the full protein structures at first, then revealed the uncompleted residues. Using the Dunbrack rotamer library, the truncated side chains were replaced with a whole side chain of the same residue type [39]. The polar hydrogens, Vina force field and Gasteiger charges were then added using VEGA [40]. Finally, the PDB protein format was converted to PDBQT. For ligand files, the VEGA was first used to prepare ligand PDBs for the same reason as the protein preparation. The pKa values of ligands were then predicted using PROPKA (version 3.1) [41,42]. MCCS would protonate the tertiary (3°) amide in the compounds when the computed pKa value of the ligands was greater than or equal to the supplied pH (7.4 by default). Finally, the torsions of ligands were determined by VEGA, and the ligand file format was changed from PDB to PDBQT. The PDBQT files of protein and ligand, as well as the pKa file of the ligand were used as input for the following phase in MCCS, which is scoring and docking with jdock (version 2.2.3b, https://github.com/stcmz/jdock accessed on 1 January 2022) [37] to prepare the complexes and to calculate the residue energy contribution.

Jdock is a core implementation of MCCS and is a version and successor of idock [43]. jdock can generate a vector of residue free energy from the conformation predicted by a Monte Carlo-based docking algorithm or determined by X-ray crystallography or cryo-EM by using the same five-term scoring function (gauss1, gauss2, repulsion, hydrophobic and h-bonding) invented by AutoDock Vina [44,45]. There are three modes: “docking” mode, “score only” mode and “scoring & docking” mode. For a given receptor–ligand complex, the scoring function can generate nine binding recognition vectors: (1) Gauss (Gauss1 + Gauss2), (2) Gauss1, (3) Gauss2, (4) repulsion, (5) steric (Gauss1 + Gauss2 + repulsion), (6) hydrogen-bonding, (7) hydrophobic, (8) non-steric (hydrogen-bonding + hydrophobic) and (9) residue energy contribution. A maximum of 999 binding poses can be generated by the docking mechanism in jdock. The X-ray crystal and cryo-EM structures were computed in “score only” mode, in which the scores of all receptor-ligand atom pairs were directly calculated and added to the overall score.

### 3.4. Molecular Dynamics (MD) Simulation and Molecular Mechanics/Generalized Born Surface Area (MM/GBSA) Calculation

Five complexes of CB2-CP55940-C-2 coupled with Gi proteins were used to perform the MD simulations, in which C-2 bound to five predicted allosteric sites. The system without C-2 binding was also prepared as a control to investigate if C-2 can enhance or decrease CP-55940 binding. Each system was put into a 0.15M NaCl solution with a cubic water box and 240 POPC lipid molecules and about 13,000 TIP3P [46] water molecules. Charmmgui (https://charmm-gui.org accessed on 1 January 2022) Online Toolkit was applied to add POPC lipids. The protein, lipid and small molecules were modeled using FF14SB [47], lipid14 [48,49], force fields, respectively. All MD simulations were performed using AMBER 18 software package [50].

Each MD system was first relaxed by five 10000-step minimizations followed by five restrained MD simulations to remove possible steric clashes. Each restrained MD simulation lasted 1 nanosecond (ns). using an integration time step of 1 femtosecond (fs). The five minimization and restrained MD runs applied 20, 10, 5, 1 and 0 kcal/mol to the mainchain atoms, sequentially. After the system was relaxed, three NPT (constant particle number, pressure and temperature) MD simulation phases were conducted sequentially: the heating-up phase (2 ns for each temperature from 50 to 250 K at a step of 50 K); the equilibrium phase (12 ns, 298 K); and the sampling phase (125 ns). The integration of the equations of motion was conducted at a time step of 2 fs for all the three phases. Other MD protocols were detailed in our previous publication [51].

250 MD snapshots were evenly selected from the sampling phase for the MM-PBSA-WSAS free energy calculations. For each MD snapshot, the molecular mechanical (MM) energy (EMM) and the Poisson–Boltzmann Surface Area (PBSA) energy terms were calculated without further minimization. Unlike the common MM-PBSA protocol for non-membrane proteins, two external dielectrics (ewat = 80 for water and elip = 1.0 for the lipid bilayer) were applied for our systems. The membrane center offset parameter (mctrdz) was calculated for an individual snapshot using the coordinate centers of CB2 receptor and the POPC bilayer. Similarly, the thickness of membrane for an individual snapshot was calculated as the distance between the centers of phosphorus atoms in the upper and lower layers of the lipids. For ligands CP55940 and C-2 themselves, the implicit membrane option was turned off and the external dielectric constant was set to 80. The nonpolar solvation energies were estimated by multiplying the solvent accessible surface areas (SAS) with surface tension coefficient plus a constant. In this work, surface tension and constant are 0.0054 kcal/(mol·Å^2^) and 0.92 kcal/mol, respectively. Note that an efficient model, WSAS, was applied to calculate the entropy contribution (TS) [52]. For MM-PBSA-WSAS binding free energy calculations, a single-trajectory protocol was applied [53], i.e., only the MD trajectories of the complex were sampled, and the receptor and ligand coordinates were extracted from the complex ones. Using this protocol, the internal energies make no contribution to the ligand binding, with a return of good error cancellation. However, in MM-PBSA-WSAS free energy calculation for the complexes, the internal energy terms (E_int_) as well as the 1–4 electrostatic (E_ele_) and van der Waals (E_VDW_) terms were all included.

## 4. Conclusions

In our previous research, we found that the binding of allosteric modulators did not cause significant impact on the conformational change for both orthosteric and allosteric binding sites of a target protein. In the present work, among seven predicted binding sites, sites B, K, G and C are supported by the known crystal structures of other class A GPCRs [14,20,21,23,24,25,26,27]. While site H has a supporting of computational and biological validation [30]. For example, we found that both site K and site B are supported by crystal structures of ADRB2 coupled with orthosteric antagonist or agonist, respectively. Site K is shown to bind with NAMs of C5aR1 human receptor and ADRB2 human receptor while site B has been supported by the ADRB2 human receptor which binds with a PAM. The crystal structure of FFaR1 human receptor–PAM complex [21] gives us another supporting that after aligning CB2 and FFaR1 human receptor, the position of PAM is inserted into site B, but the tail of the PAM is connected to site K. Thus, site B is more likely to bind with PAMs, while sites C, G and K have higher possibility to bind with NAMs. However, based on the results of MD simulations, site H is the most possible binding site of CB2 for the allosteric binding of C-2. It may because site H is adjacent to the orthosteric site and C-2 can directly have a favorable interaction with CP55940. Among the residues of site H, H95^2.65^ and S285^7.38^ may be the important binding residues that form hydrogen bonds with C-2. The H95^2.65^ and S285^7.38^ in CB2 refer to H178^2.65^ and S383^7.38^ in CB1, respectively. Thinking about the selectivity in receptor subtypes, if these residues are key binding residues, CB1 may form the same interactions (hydrogen bonds) as CB2, which is not ideal for designing an AM that selectively targeted on CB2. Ligand selectivity is always a challenging problem for researchers to understand or to deal with, not only for orthosteric ligands but also for AMs [54]. So much more efforts should be taken to study, compare and analyze the high-resolution structures of CB1 and CB2. Taking the supporting crystal structures (e.g., ADRB2 human receptor) into consideration, the allosteric modulators with different properties may have different binding sites. The potential key residues in each site, especially for those supported by the crystal structures, provide a new sight on designing unique AMs for specific binding sites.

## Figures and Tables

**Table 1:** The comparisons of docking scores of AMs in various allosteric binding sites (kcal/mol).

	Site B	Site C	Site G	Site H	Site I	Site J	Site K
C−2	−7.26	N/A *	−6.68	−7.20	−7.31	−5.64	−6.95
TBC	−6.04	−4.44	−5.42	−2.79	−6.39	−4.35	−5.75
CBD	−6.28	−4.74	−5.89	−4.21	−5.92	−4.13	−5.78

* N/A: based on the reported crystal structures, we suggested that C-2 (PAM) did not bind to site C.

**Table 2:** Energy decomposition of MM-PBSA-WSAS free energy for the CB2/CP55940/C-2 complex. All energies are in kcal/mol.

Site	E_VDW_	E_eel_	E_int_	G_pol_	G_nonpol_	TS	G_MM-PBSA_
B	−1213.52 ± 1.02	−6211.37 ± 5.31	7207.92 ± 5.38	−3699.43 ± 2.93	87.94 ± 0.14	3406.36 ± 0.68	−7234.83 ± 8.24
G	−1231.75 ± 0.80	−6271.31 ± 10.20	7216.25 ± 6.29	−3646.51 ± 16.49	89.27 ± 0.10	3407.79 ± 0.35	−7251.85 ± 1.51
H	−1249.43 ± 3.56	−6356.62 ± 3.06	7216.04 ± 2.52	−3623.11 ± 3.62	85.70 ± 0.01	3393.90 ± 0.08	−7321.31 ± 7.11
I	−1257.82 ± 1.33	−6363.47 ± 9.32	7204.73 ± 4.78	−3520.82 ± 6.20	86.77 ± 0.07	3395.35 ± 0.13	−7245.96 ± 11.47
J	−1224.81 ± 1.81	−6261.38 ± 3.18	7224.34 ± 5.16	−3738.61 ± 7.43	87.58 ± 0.04	3401.05 ± 0.31	−7313.93 ± 4.07

E_VDW_: Van der Waals energy; E_eel_: Electrostatic energy; E_int_: Internal energy; G_pol_: the polar part of solvation free energy; G_nonpol_: the nonpolar part of solvation free energy; TS: the entropy contribution of the MM-PBSA free energy; G_MM-PBSA_: MM-PBSA free energy.

**Table 3:** Energy decomposition of MM-PBSA-WSAS free energy for C-2 binding to CB2 with CP55940 residing in the orthosteric site. All energies are in kcal/mol.

Site	ΔE_VDW_	ΔE_eel_	ΔG_pol_	ΔG_nonpo_	TΔS	ΔG_MM-PBSA_
B	−27.47 ± 0.49	2.42 ± 0.18	7.11 ± 0.31	−2.65 ± 0.03	−17.80 ± 0.09	−2.79 ± 0.64
G	−28.83 ± 0.22	−3.92 ± 0.21	12.03 ± 0.43	−2.30 ± 0.02	−17.36 ± 0.15	−5.66 ± 0.68
H	−56.18 ± 0.08	−24.69 ± 0.50	24.41 ± 0.19	−3.79 ± 0.01	−26.13 ± 0.08	−34.12 ± 0.43
I	−32.57 ± 0.08	−3.01 ± 0.06	10.97 ± 0.08	−2.58 ± 0.02	−18.47 ± 0.07	−8.73 ± 0.12
J	−25.14 ± 0.41	−9.96 ± 0.73	23.44 ± 0.55	−2.15 ± 0.03	−17.38 ± 0.17	3.57 ± 0.44

E_VDW_: Van der Waals energy; E_eel_: Electrostatic energy; G_pol_: the polar part of solvation free energy; G_nonpol_: the nonpolar part of solvation free energy; TΔS: the entropy contribution for ligand binding at temperature T; G_MM-PBSA_: MM-PBSA free energy.

**Table 4:** Energy decomposition of MM-PBSA-WSAS free energy for CP55940 binding to CB2 with C-2, an allosteric agonist existing. All energies are in kcal/mol. H* implies that the C-2 is considered as a part of the receptor.

Site	ΔE_VDW_	ΔE_eel_	ΔG_pol_	ΔG_nonpo_	TΔS	ΔG_MM-PBSA_
No Ligand	−53.69 ± 0.21	−18.76 ± 0.18	24.97 ± 0.27	−4.88 ± 0.01	−24.96 ± 0.07	−27.39 ± 0.23
B	−56.87 ± 0.21	−20.24 ± 0.65	25.14 ± 0.27	−4.96 ± 0.00	−25.47 ± 0.02	−31.47 ± 0.18
G	−58.05 ± 0.25	−9.74 ± 0.36	23.54 ± 0.19	−4.96 ± 0.01	−25.56 ± 0.09	−23.66 ± 0.50
H	−56.21 ± 0.27	−11.57 ± 0.40	21.30 ± 0.15	−4.91 ± 0.01	−25.31 ± 0.04	−26.09 ± 0.01
H*	−60.94 ± 0.34	−12.78 ± 0.38	20.00 ± 0.19	−4.88 ± 0.02	−26.55 ± 0.05	−32.04 ± 0.29
I	−57.95 ± 0.21	−18.73 ± 0.23	23.42 ± 0.11	−4.77 ± 0.01	−26.20 ± 0.03	−31.83 ± 0.15
J	−57.69 ± 0.13	−26.88 ± 0.50	24.26 ± 0.16	−4.80 ± 0.01	−26.81 ± 0.06	−38.29 ± 0.40

E_VDW_: Van der Waals energy; E_eel_: Electrostatic energy; G_pol_: the polar part of solvation free energy; G_nonpol_: the nonpolar part of solvation free energy; TΔS: the entropy contribution for ligand binding at temperature T; G_MM-PBSA_: MM-PBSA free energy.

## Data Availability

Data is contained within the article.

## Sample Availability

Samples of the compounds mentioned in the paper are not available from the authors.
